# Seasonality Affects Elderly Hip Fracture Mortality Risk During the COVID-19 Pandemic

**DOI:** 10.7759/cureus.26530

**Published:** 2022-07-03

**Authors:** Garrett W Esper, Ariana T Meltzer-Bruhn, Abhishek Ganta, Kenneth A Egol, Sanjit R Konda

**Affiliations:** 1 Orthopedic Surgery, New York University Langone Health, New York, USA; 2 Orthopedic Surgery, State University of New York Upstate Medical University, Syracuse, USA; 3 Medical School, University of Pennsylvania Perelman School of Medicine, Philadelphia, USA; 4 Orthopedic Surgery, Jamaica Hospital Medical Center, New York, USA; 5 Orthopaedic Surgery, Jamaica Hospital Medical Center, New York, USA

**Keywords:** covid-19, risk stratification, geriatric, seasonality, hip fracture

## Abstract

Background

The incidence of geriatric hip fractures, respiratory infections (e.g., coronavirus disease 2019 (COVID-19), influenza), and mortality is higher during the fall and winter. The purpose of this study is to assess whether the addition of seasonality to a validated geriatric inpatient mortality risk tool will improve the predictive capacity and risk stratification for geriatric hip fracture patients. We hypothesize that seasonality will improve the predictive capacity.

Methodology

Between October 2014 and August 2021, 2,421 patients >55-year-old treated for hip fracture were analyzed for demographics, date of presentation, COVID-19 status (for patients after February 2020), and mortality. Patients were grouped by season based on their admission dates into the following four cohorts: fall (September-November), winter (December-February), spring (March-May), and summer (June-August). Patients presenting during the fall/winter and spring/summer were compared. The baseline Score for Trauma Triage in the Geriatric and Middle-Aged (STTGMA) tool for hip fractures (STTGMAHIP_FX_SCORE) and the seasonality iteration (STTGMA_SEASON) were also compared. Sub-analysis was conducted on 687 patients between February 2020 and August 2021 amid the COVID-19 pandemic. The baseline score (STTGMAHIP_FX_SCORE) and the COVID-19 iteration (STTGMACOVID_ORIGINAL_2020) were modified to include seasonality (STTGMA_COVID/SEASON). Patients were stratified by risk score and compared. The predictive ability of the models was compared using DeLong’s test.

Results

For the overall cohort, patients who presented during the fall/winter had a higher rate of inpatient mortality (2.87% vs. 1.25%, p < 0.01). STTGMA_SEASON improved the predictive capacity for inpatient mortality compared to STTGMAHIP_FX_SCORE but not significantly (0.773 vs. 0.672, p = 0.105) On sub-analysis, regression weighting showed a coefficient of 0.643, with fall and winter having a greater absolute effect size (fall = 2.572, winter = 1.929, spring = 1.286, summer = 0.643). STTGMA_COVID/SEASON improved the predictive capacity for inpatient mortality compared to STTGMAHIP_FX_SCORE (0.882 vs. 0.581, p < 0.01) and STTGMACOVID_ORIGINAL_2020 (0.882 vs. 0.805, p = 0.04). The highest risk quartile contained 89.5% of patients who expired during their index inpatient hospitalization (p < 0.01) and 68.2% of patients who died within 30 days of discharge (p < 0.01).

Conclusions

Seasonality may play a role in both the incidence and impact of COVID-19 and additional respiratory infections. Including seasonality improves the predictive capacity and risk stratification of the STTGMA tool during the COVID-19 pandemic. This allows for effective triage and closer surveillance of high-risk geriatric hip fracture patients by better accounting for the increased respiratory infection incidence and the associated mortality risk seen during fall and winter.

## Introduction

The incidence of geriatric hip fractures, respiratory infections (e.g., coronavirus disease 2019 (COVID-19), influenza, etc.), and mortality is higher during the fall and winter [1-5]. Geriatric individuals have waning immune responses due to aging and chronic comorbidity that makes them more susceptible to various infections, especially respiratory tract infections such as community and hospital-acquired pneumonia [6,7]. In the United States, from 1999 to 2020, pneumonia and influenza were the 10th leading cause of death in people 65 years of age and older and respiratory infections were the leading cause of death due to infection in the elderly [8,9]. With the emergence of the COVID-19 virus in early 2020, COVID-19 has become the third leading cause of death in individuals aged 65 and older [9]. Similar to other respiratory infections, the seasonal variation in COVID-19 surges is an important factor to consider when assessing the higher rates of mortality seen in the geriatric population in the winter months [10].

Various studies across the globe, including in the United Kingdom, United States, Europe, and Egypt, have demonstrated an increase in the number of geriatric hip fractures that occur during the winter months and periods of colder temperatures [1-5]. The cause of this is multifactorial: higher fall rates due to harsher outdoor conditions (ice, snow, etc.) and increased bone fragility in older patients due to seasonal variation in parathyroid hormone and vitamin D levels [1,11,12]. Various studies have also demonstrated a higher mortality rate associated with hip fractures in geriatric patients during the winter months [2,4,13,14]. Existing literature attributes some of the increased mortality to a worsening of pre-existing pulmonary and cardiovascular conditions and higher rates of pulmonary infections in geriatric patients during winter [13,15].

As hip fractures in the geriatric population carry a significantly high rate of morbidity and mortality at baseline, the effective care and treatment of these patients are of great importance to healthcare providers [16]. Risk profiling these patients upon hospital admission helps providers develop appropriate treatment plans to meet their care needs. The Score for Trauma Triage in the Geriatric and Middle-Aged (STTGMA) is an inpatient mortality risk assessment tool validated in patients aged 55 and older who sustain orthopedic trauma injuries [17]. The original STTGMA tool utilized clinical data available in the emergency department (ED) setting to calculate a mortality risk score on arrival at the hospital. Variables included in the original iteration were a patient’s age, injury details, Glasgow Coma Scale (GCS) score, and comorbidity profile based on Charlson Comorbidity Index (CCI) [17]. Since its inception, the STTGMA tool has evolved through several iterations to now include variables such as ambulatory status, a patient’s American Society of Anesthesiologists (ASA) score, and their COVID-19 status on admission [18-20].

Given the STTGMA tool’s adaptive capability to improve its predictive capacity, the purpose of this study is to determine whether the inclusion of a seasonality factor would further improve the predictive capacity and risk stratification for geriatric hip fracture patients. Given the current state of affairs with the COVID-19 pandemic, this study examines the inclusion of a seasonality factor for both 2014 to 2021 as well as just for the COVID-19 pandemic. We hypothesize that seasonality will improve the predictive capacity of the STTGMA tool.

## Materials and methods

For this retrospective cohort study, an Institutional Review Board-approved trauma database was reviewed and queried for all patients aged 55 or older who sustained a hip fracture through a low-energy mechanism (low energy is defined as a fall from standing or from a height fewer than two stairs) between October 2014 and August 2021. All patients were treated at one academic medical center including three level 1 trauma centers, one university-based tertiary care referral hospital, and one orthopedic specialty hospital. Patients were included if they sustained a subtrochanteric, femoral neck, or intertrochanteric hip fracture (AO Foundation/Orthopaedic Trauma Association fracture classifications: 31A, 31B, 32(A-C)). Patients were excluded if they were younger than 55 years of age or had a high-energy mechanism of injury.

Each patient chart was reviewed for demographics including age, body mass index (BMI), sex, baseline ambulatory status, and comorbidities (compiled via the CCI). Injury presentation variables collected were the GCS score and the Abbreviated Injury Severity scores (AIS) for both the Head/Neck (AIS H/N) and Chest (AIS C). Hospital quality measures collected included the length of stay (LOS), the need for intensive care unit (ICU) level of care, and discharge location. Discharge home was defined as either home independently or home with a health service aide. Mortality measures collected included inpatient mortality and mortality within 30 days of discharge. Inpatient complications recorded during each patient’s index hospitalization included sepsis/septic shock, pneumonia, deep vein thrombus/pulmonary embolism (DVT/PE), myocardial infarction (MI), acute kidney injury (AKI), stroke, surgical site infection (SSI), decubitus ulcer, urinary tract infection (UTI), acute respiratory failure (ARF), anemia, and cardiac arrest.

To demonstrate a similar cohort to historical literature, patients were initially grouped based on their time of presentation: during the cold seasons (September-February or fall/winter) or warm seasons (March-August or spring/summer). Comparative analyses were conducted between these two cohorts. Next, the baseline STTGMA tool for hip fractures (STTGMAHIP_FX_SCORE) was calculated for each patient.

A second analysis was conducted with patients grouped by season based on their admission dates: fall (September-November), winter (December-February), spring (March-May), and summer (June-August). The STTGMA models were then adapted to include this seasonality factor (STTGMA_SEASON), and new mortality risk scores were calculated for each patient.

A sub-analysis was conducted on 687 patients who presented during the COVID-19 pandemic between February 2020 and August 2021. Both the baseline STTGMA score (STTGMAHIP_FX_SCORE) and the COVID-19 iteration (STTGMA_COVID_ORIGINAL_2020) were calculated for each patient [20]. Patients were again grouped by season into four cohorts. The STTGMA model was adapted to include the seasonality factor (STTGMA_COVID/SEASON), and new mortality risk scores were calculated for each patient.

Each model’s predictive ability was compared using DeLong’s test to analyze the area under the receiver operating curves (AUROCs). Patients were stratified into risk quartiles based on their new respective STTGMA_COVID/SEASON mortality risk score. Comparative analyses were conducted on each risk quartile to compare the effectiveness of this seasonality inclusion.

Mann-Whitney U tests, chi-square tests, independent-sample t-tests, and analysis of variance (ANOVA) were used as appropriate. Statistics were calculated using SPSS software version 25 (IBM Corp., Armonk, NY, USA). Significance was defined with an alpha of 0.05.

## Results

In total, 2,421 patients met the inclusion criteria for the 2014-2021 full cohort. Overall cohort characteristics were 69% female, mean age of 80.7 ± 10.2 years, mean BMI of 24.17 ± 4.94, mean GCS of 14.87 ± 0.63, mean CCI of 1.49 ± 1.73, mean AIS Head/Neck of 0.03 ± 0.27, and a mean AIS Chest of 0.02 ± 0.19. Most patients were White (71.71%). At baseline, the majority of patients were community ambulators (67.91%), while 28.17% of patients were household ambulators and 3.92% were non-ambulatory (Table 1).

**Table 1:** Demographic breakdown of the overall cohort.

Cohort Demographics	Total n (%)
N	2,421
Age	80.70 ± 10.20
Body mass index	24.17 ± 4.94
Charlson Comorbidity Index	1.49 ± 1.73
Male	739 (30.52%)
Female	1,682 (69.48%)
White	1,736 (71.71%)
Black	190 (7.85%)
Hispanic	131 (5.41%)
Asian	202 (8.34%)
Other	48 (1.98%)
Unknown	114 (4.71%)
Community ambulatory	1,644 (67.91%)
Household ambulatory	682 (28.17%)
Non-ambulatory/Wheelchair	95 (3.92%)
Glasgow Coma Scale	14.87 ± 0.63
Abbreviated Injury Score Head/Neck	0.03 ± 0.27
Abbreviated Injury Score Chest	0.02 ± 0.19

When comparing initial cohorts based on historical literature (cold seasons vs. warm seasons), patients who sustained their injuries and presented during fall/winter experienced higher rates of ARF (5.75% vs. 3.91%, p = 0.037), cardiac arrest (1.64% vs. 0.75%, p = 0.044), and inpatient mortality (2.87% vs. 1.25%, p < 0.01) (Table 2).

**Table 2:** Comparison of outcomes between patients presenting in the cold (fall/winter) and warm (spring/summer) seasons for the overall cohort (2014-2021). DVT/PE = deep vein thrombosis/pulmonary embolism; MI = myocardial infarction; AKI = acute kidney injury; SSI = surgical site infection; UTI = urinary tract infection; ARF = acute respiratory failure; LOS = length of stay; SD = standard deviation; ICU = intensive care unit

Outcomes	Fall/Winter n (%)	Spring/Summer n (%)	Total n (%)	P-value
N	1,218	1,203	2,421	
Sepsis/Septic shock	34 (2.79%)	20 (1.66%)	54 (2.23%)	0.062
Pneumonia	64 (5.25%)	50 (4.16%)	114 (4.71%)	0.207
DVT/PE	29 (2.38%)	18 (1.50%)	47 (1.94%)	0.118
MI	16 (1.31%)	14 (1.16%)	30 (1.24%)	0.748
AKI	102 (8.37%)	100 (8.31%)	202 (8.34%)	0.981
Stroke	6 (0.49%)	5 (0.42%)	11 (0.45%)	0.782
SSI	1 (0.08%)	3 (0.25%)	4 (0.17%)	0.309
Decubitus ulcer	13 (1.07%)	20 (1.66%)	33 (1.36%)	0.203
UTI	103 (8.46%)	87 (7.23%)	190 (7.85%)	0.273
ARF	70 (5.75%)	47 (3.91%)	117 (4.83%)	0.037
Anemia	394 (32.35%)	350 (29.09%)	744 (30.73%)	0.093
Cardiac arrest	20 (1.64%)	9 (0.75%)	29 (1.20%)	0.044
LOS (d, mean ± SD)	6.66 ± 4.52	6.34 ± 4.36	6.50 ± 4.44	0.519
Need for ICU	222 (18.23%)	232 (19.29%)	454 (18.75%)	0.505
Discharged home	272 (22.33%)	307 (25.52%)	579 (23.92%)	0.066
Inpatient mortality	35 (2.87%)	15 (1.25%)	50 (2.07%)	<0.01
30-day mortality	62 (5.09%)	51 (4.24%)	113 (4.67%)	0.321

**Figure 1:**
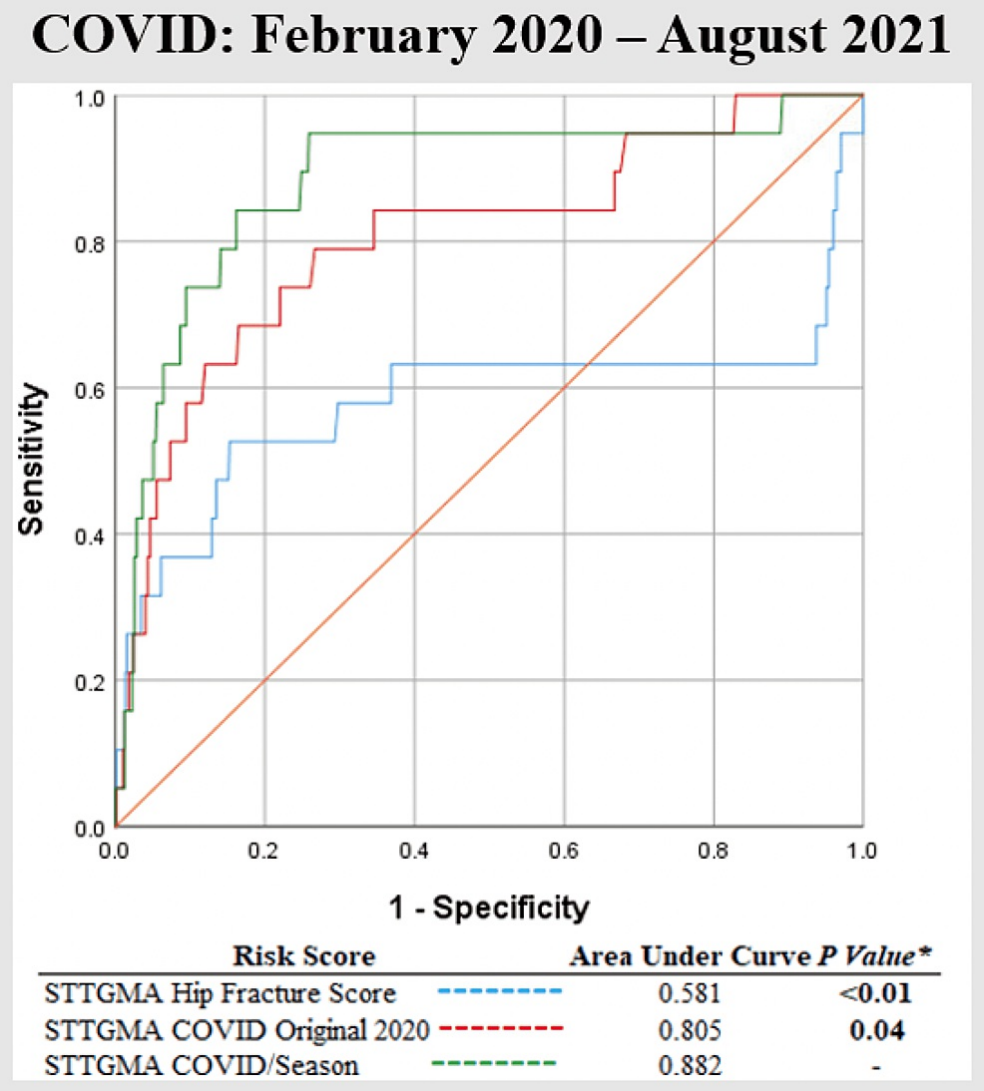
Comparison of the AUROCs for the STTGMAHIP_FX_SCORE, STTGMA_COVID_ORIGINAL_2020, and STTGMA_COVID/SEASON mortality risk scores. AUROC = area under receiver operating curves; STTGMA = Score for Trauma Triage in the Geriatric and Middle-Aged

AUROC comparison for the overall cohort demonstrated STTGMA_SEASON improved the predictive capacity for inpatient mortality compared to STTGMAHIP_FX_SCORE; however, it was not significant (0.773 vs. 0.672, p = 0.105). On sub-analysis, AUROC comparison demonstrated STTGMA_COVID/SEASON significantly improved the predictive capacity for inpatient mortality compared to STTGMAHIP_FX_SCORE (0.882 vs. 0.581, p < 0.01) and STTGMA_COVID_ORIGINAL_2020 (0.882 vs. 0.805, p = 0.04) (Figure 1).

Regression weighting showed a coefficient of 0.643, with fall and winter having a greater absolute effect size (fall = 2.572, winter = 1.929, spring = 1.286, summer = 0.643).

When comparing risk quartiles for STTGMA_COVID/SEASON, the highest risk quartile contained 89.5% of patients who died during their index inpatient hospitalization (p < 0.01) and 68.2% of patients who died within 30 days of hospital discharge (p < 0.01). Additionally, patients in the high-risk cohort experienced a longer LOS (p < 0.01), higher rates of sepsis (p < 0.01), pneumonia (p < 0.01), DVT/PE (p = 0.01), ARF (p < 0.01), anemia (p < 0.01), cardiac arrest (p < 0.01), need for ICU level of care (p < 0.01), and were the least likely to be discharged home (p < 0.01) (Table 3). 

**Table 3:** Comparison of outcomes during the pandemic (2020-2021) between risk quartiles based on STTGMA_COVID/SEASON mortality risk score. DVT/PE = deep vein thrombosis/pulmonary embolism; SSI = surgical site infection; UTI = urinary tract infection; ARF = acute respiratory failure; LOS = length of stay; ICU = intensive care unit; STTGMA = Score for Trauma Triage in the Geriatric and Middle-Aged

STTGMA risk score	>2.37%	2.36%-1.12%	1.11%-0.61%	<0.61%	
N	171	172	172	172	
Sepsis/Septic shock	11 (6.43%)	2 (1.16%)	2 (1.18%)	0 (0.00%)	<0.01
Pneumonia	27 (15.79%)	2 (1.16%)	4 (2.33%)	3 (1.74%)	<0.01
DVT/PE	7 (4.09%)	2 (1.16%)	2 (1.16%)	1 (0.58%)	0.010
Myocardial infarction	4 (2.34%)	3 (1.74%)	0 (0.00%)	1 (0.58%)	0.110
Acute kidney injury	22 (12.87%)	22 (12.79%)	15 (8.72%)	5 (2.91%)	0.070
Stroke	2 (1.17%)	0 (0.00%)	1 (0.58%)	1 (0.58%)	0.230
SSI	1 (0.58%)	1 (0.58%)	0 (0.00%)	1 (0.58%)	0.799
Decubitus ulcer	2 (1.17%)	2 (1.16%)	3 (1.74%)	1 (0.58%)	0.799
UTI	10 (5.85%)	10 (5.81%)	10 (5.81%)	5 (2.91%)	0.518
ARF	21 (12.28%)	7 (4.07%)	6 (3.49%)	1 (0.58%)	<0.01
Anemia	62 (36.26%)	47 (27.33%)	47 (27.33%)	24 (13.95%)	<0.01
Cardiac arrest	8 (4.68%)	1 (0.58%)	1 (0.58%)	0 (0.00%)	<0.01
LOS (d, mean ± SD)	7.15 ± 6.22	5.67 ± 3.47	5.66 ± 3.75	5.09 ± 3.60	<0.01
Need for ICU	54 (31.58%)	25 (14.45%)	29 (17.06%)	21 (12.14%)	<0.01
Discharged home	34 (19.88%)	42 (24.28%)	56 (32.94%)	77 (44.51%)	<0.01
Inpatient mortality	17 (9.94%)	1 (0.58%)	0 (0.00%)	1 (0.58%)	<0.01
30-day mortality	30 (17.54%)	9 (5.20%)	3 (1.76%)	2 (1.16%)	<0.01

## Discussion

The purpose of this study was to assess if the addition of a seasonality factor to a validated geriatric inpatient mortality risk tool improved the predictive capacity and risk triage for geriatric hip fracture patients. While seasonality did not significantly increase the predictive capacity for the overall cohort, this study demonstrates that the inclusion of seasonality allows for improved modeling amid the COVID-19 pandemic. This new factor may allow for more effective triage and risk stratification to better account for the higher mortality rate seen during the fall and winter seasons. This may be due to accounting for the higher rates of respiratory infection that naturally occur in the fall and winter months, compounded by the recent COVID-19 surges.

Our study demonstrates that geriatric patients who sustained their injury and presented during the fall and winter seasons had a higher risk of inpatient mortality. This aligns with research seen in current literature that found a higher mortality rate in patients who sustained a hip fracture during the colder fall and winter seasons [1-5]. While our study does not specifically investigate the cause of this increased mortality, the higher rates of ARF and cardiac arrest may provide some insights into possible causes. As seen in the literature, geriatric patients experience a worsening of their baseline comorbid conditions during the fall and winter months [13,15]. The immunosenescence seen in the elderly along with the worsening of their baseline health in winter may explain the elevated mortality risk [13,15,21]. While our study does account for the baseline comorbidity of each patient through the CCI, this does not capture a “worsening” of these conditions that may be seen. Similarly, our study did not capture possible infection with other respiratory infections apart from COVID-19 at the time of their injury. However, as patients in the cold weather cohort did experience a higher rate of pneumonia as an inpatient complication (albeit not significantly) and a higher rate of ARF, the role of respiratory infection in this elevated mortality rate is very possible. Therefore, it is important for providers involved in the care of geriatric patients to keep these elevated mortality risks in mind, especially in the winter months.

The role of seasonality in the incidence of respiratory infections, most commonly influenza, is well documented [22]. This is due to both environmental factors and patient/societal behavior [23]. More recently, the onset and continuation of the COVID-19 pandemic have demonstrated significant seasonal variation in incidence and severity [24]. Similar to influenza, environmental factors such as temperature and humidity play a role in the seasonality of COVID-19 infections [25]. Behavioral factors such as fewer outdoor activities in fall and winter may also play a role, with airborne transmission being the most common pathway, especially in close environments with minimal wind and air movement [24,26]. While societal interventions such as social distancing and mask-wearing guidelines have attempted to combat these infection risk factors, it is important to consider the role of seasonality when constructing care models for geriatric patients at high risk for poor outcomes as infection numbers have continued to fluctuate.

While the addition of the seasonality factor improved our model’s predictive ability for the overall cohort from 2014 to 2021, the change in the area under the curve was not significant, as determined by Delong’s test. However, on a sub-analysis of a COVID-19 pandemic cohort, the model’s predictive capacity improved significantly. This is likely due to the pronounced increase in mortality risk associated with COVID-19 [27,28]. Therefore, with COVID-19 surges occurring predominantly in the fall and winter seasons, as discussed above, our seasonality factor accounts for this newfound respiratory infection burden in addition to previous respiratory infections (influenza) and the worsening of baseline comorbidities during the fall and winter seasons. Usage of the STTGMA_COVID/SEASON tool on our patient population allowed for significant triage into risk quartiles. This allows for high-risk patients to be recognized on arrival at the ED and for more intensive medical optimization and timely surgical intervention to be provided if required. These risk factors are important for healthcare teams to be aware of and use in care modulation. For example, long-term care facilities often have high rates of respiratory infection outbreaks [29]. In fall and winter, healthcare teams may target discharge home for patients in the highest risk groups instead of a skilled nursing facility to help minimize infection and subsequent mortality risk. Likewise, patients with minimal risk can also be targeted for discharge home, reducing the burden on skilled nursing and acute rehabilitation facilities. Downstream, this may help lower the risk of further morbidity and mortality associated with discharge to these facilities which has been demonstrated during the COVID-19 pandemic.

This study has several limitations. Being based in New York, our institution’s location and experience of four complete seasons may limit the efficacy of these factors in different regions that do not have the same seasonal variation. While past iterations of the STTGMA tool included variables unaffected by geographic location, the same cannot be said for the seasonality factor. Additional studies may be conducted in the future at different geographic locations to allow for the seasonality factor to be adapted for different regions. Future studies may also include a cost analysis to assess seasonal variation in admission costs for hip fracture patients. Lastly, future studies may be focused on further investigation of the underlying causes of increased mortality seen in the geriatric population in fall and winter.

## Conclusions

Seasonality may play a role in both the incidence and impact of COVID-19 and additional respiratory infections. Including seasonality improves the predictive capacity and risk stratification of STTGMA during COVID-19. This allows for effective triage and closer surveillance of high-risk geriatric hip fracture patients by better accounting for the increased respiratory infection incidence and associated mortality risk seen during fall and winter.
